# Effects of Poor Maternal Nutrition during Gestation on Bone Development and Mesenchymal Stem Cell Activity in Offspring

**DOI:** 10.1371/journal.pone.0168382

**Published:** 2016-12-12

**Authors:** Sambhu M. Pillai, Nicole H. Sereda, Maria L. Hoffman, Ellen V. Valley, Thomas D. Crenshaw, Young-Ki Park, Ji-Young Lee, Steven A. Zinn, Kristen E. Govoni

**Affiliations:** 1 Department of Animal Science, University of Connecticut, Storrs, Connecticut, United States of America; 2 Department of Animal Sciences, University of Wisconsin, Madison, Wisconsin, United States of America; 3 Department of Nutritional Sciences, University of Connecticut, Storrs, Connecticut, United States of America; Rutgers New Jersey Medical School, UNITED STATES

## Abstract

Poor maternal nutrition impairs overall growth and development of offspring. These changes can significantly impact the general health and production efficiency of offspring. Specifically, poor maternal nutrition is known to reduce growth of bone and muscle, and increase adipose tissue. Mesenchymal stem cells (MSC) are multipotent stem cells which contribute to development of these tissues and are responsive to changes in the maternal environment. The main objective was to evaluate the effects of poor maternal nutirtion during gestation on bone and MSC function in offspring. Thirty-six ewes were fed 100%, 60%, or 140% of energy requirements [NRC, 1985] beginning at day 31 ± 1.3 of gestation. Lambs from ewes fed 100% (CON), 60% (RES) and 140% (OVER) were euthanized within 24 hours of birth (1 day; n = 18) or at 3 months of age (n = 15) and bone and MSC samples were collected. Dual X-ray absorptiometry was performed on bones obtained from day 1 and 3 months. Proliferation, differentiation, and metabolic activity were determined in the MSC isolated from lambs at day 1. Data were analyzed using mixed procedure in SAS. Maternal diet negatively affected offspring MSC by reducing proliferation 50% and reducing mitochondrial metabolic activity. Maternal diet did not alter MSC glycolytic activity or differentiation in culture. Maternal diet tended to decrease expression of P2Y purinoreceptor 1, but did not alter expression of other genes involved in MSC proliferation and differentiation. Maternal diet did not alter bone parameters in offspring. In conclusion, poor maternal diet may alter offspring growth through reduced MSC proliferation and metabolism. Further studies evaluating the potential molecular changes associated with altered proliferation and metabolism in MSC due to poor maternal nutrition are warranted.

## Introduction

In multiple species, poor maternal nutrition during gestation impairs fetal and postnatal growth and development. However, the specific mechanisms are not clear [1,2]. Poor maternal nutrition can result from excess or reduced nutrient intake including energy, protein, and micronutrients in the diet. Poor maternal nutrition during gestation is known to reduce fetal growth [2–4], impair muscle development [5,6], reduce bone density [1,7], increase fat accretion [5,8,9], alter metabolism [2,3,10], and impair stem cell function [11,12] in the offspring.

Previous work by our laboratory group and others demonstrates that poor maternal nutrition alters muscle, bone, and fat development in offspring during pre- and post-natal growth. Specifically, using a sheep model, restricted- and over-feeding during gestation altered muscle cross sectional area in offspring at birth and three months of age, and increased adipose content of muscle [6]. Additionally, subcutaneous fat was reduced in offspring from restricted-fed ewes [13]. The known effects of maternal diet on fetal and postnatal bone of the offspring are limited; however, maternal diet may program offspring for reduced bone later in life [14]. Poor maternal nutrition during gestation not only has a direct effect on offspring nutrient availability, but also creates an environment of stress that can lead to long-term or permanent effects on tissue development and stem cell function [5,14]. Thus, the negative effects on offspring are well documented, but mediators of these effects are not well established.

Mesenchymal stem cells are multipotent stem cells that contribute to the development of several tissues of mesenchyme origin, including muscle, bone, and adipose, as well as maintenance and repair of these tissues through adulthood [15]. Additionally, MSC are key components of the bone marrow niche, which are responsive to hormonal and metabolic changes in the whole body [16]. Factors, such as maternal diet, may initiate MSC to differentiate into one lineage versus another (e.g., adipose vs. bone) [14]. This diversion could be a potential mechanism by which poor maternal diet alters the development of muscle and adipose tissue as well as bone. Furthermore, this may contribute to the increased risk of obesity and osteoporosis later in life in the offspring [14]. For example, in a rodent model, MSC of offspring from mothers fed a low protein diet during pregnancy exhibited reduced proliferation and ability to differentiate into bone forming cells (osteoblasts) at 8 weeks of age [11]. Maternal programming of MSC may contribute to altered muscle, bone, and adipose tissue growth in offspring. However, the specific effects of poor maternal nutrition due to restricted- or over-feeding on MSC function and metabolism need to be evaluated. The objective of the current study was to determine the effects of poor maternal nutrition on bone development and MSC proliferation, differentiation and energy metabolism in offspring at birth and at three months of age. We hypothesized that poor maternal nutrition during gestation would reduce bone density and impair MSC function and metabolism in the offspring.

## Materials and Methods

### Animals

All animal protocols were reviewed and approved by the University of Connecticut Institutional Animal Care and Use Committee. Thirty-six multiparous ewes from the University of Connecticut sheep flock (25 Dorsets, 7 Shropshires, and 4 Southdowns) were estrus synchronized [17] with progesterone controlled intravaginal drug release devices (Pfizer Animal Health; New York, NY, USA) and Lutaylase (Pfizer Animal Health). These estrus synchronized ewes were bred by live cover to one ram of like breed (3 Dorsets rams, 1 Southdown ram, and 1 Shropshire ram) as previously described [6,12,13]. Date of breeding was considered the day that the ewes were marked by the ram. Pregnancy was confirmed by ultrasound by 30 days of gestation and ewes were moved into individual pens. Ewes were randomly assigned to 1 of 3 diets: 100%, 60% or 140% NRC requirements for energy requirements (total digestible nutrients) and remained on study until they gave birth [6]. Lambs from control-fed (**CON**), over-fed (**OVER**) and restricted-fed ewes (**RES**) remained with their mother to ensure adequate colostrum intake for up to 24 hours. One lamb from each ewe was removed and euthanized at either 1 day or 3 months of age. Lambs raised until 3 months of age were fed milk replacer (1.7% of BW; Land O’Lakes Animal Milk Product Company; Shoreview, MN) from a bottle until weaning at 60 days of age. They were allowed ad libitum access to water, creep feed (Lamb BT, Blue Seal Feeds; Litchfield, CT), and second cutting hay for the entire three month period [6]. A total of 33 lambs were used for the study at 1 day (n = 18) and 3 months (n = 15). Information regarding the offspring and gender distribution and selection criterion for offspring have been described in detail [6].

### Sample Collection and Analysis

Animals were euthanized with an intravenous injection of Beuthanasia-D Special (Merck Animal Health; Summit, NJ, USA) containing 390 mg/mL sodium pentobarbital and 50 mg/mL phenytoin based on BW (0.22 mL/kg), followed by exsanguination, before performing necropsy. At necropsy, femur and tibia bones were collected after removing the wool, skin, and muscles. The femur and tibia from the right leg (1 day, n = 6/treatment; 3 months, n = 4-5/treatment) were used for determining bone mineral content (BMC), bone area and bone mineral density (BMD) using dual-energy X-ray absorptiometry (DXA; software version 12.20; GE Lunar Prodigy, Waukesha, WI). Bone marrow MSC were isolated from femur and tibia from the left leg of offspring at 1 day (n = 6/treatment) as previously described [18]. Briefly, bones were soaked with PBS and transported to the laboratory. Bones were then rinsed with 70% ethanol and both ends of the bones were removed. In a sterile hood, the interior of the bone was rinsed with α-MEM (Life Technologies, Carlsbad, CA, USA) into a cell culture dish and collected in a Falcon tube (Thermo Fisher Scientific; Waltham, MA, USA) for centrifugation. Bone marrow was centrifuged for three minutes at 1,000 × *g*. Ammonium chloride (Stem Cell Technologies; Vancouver, BC, Canada) was used to lyse red blood cells (RBC). Bone marrow MSC were isolated after filtering the lysed RBC through a 70 μm pore filter. Cells were counted using a hemocytometer and plated at 12 to 15 million cells per cell culture dish (100 x 20 mm; Greiner Bio-One; Monroe, NC, USA) in maintenance media that consisted of α-MEM (Life Technologies, Carlsbad, CA, USA), 10% fetal bovine serum (FBS; Atlanta Biologicals; Charlotte, NC, USA), 0.5% Penicillin-Streptomycin and 0.25% Fungizone. Cells were incubated at 37°C and 5% CO_2_ for cell expansion with media changed every two to three days. When the adherent cells were 70 to 80% confluent, they were frozen using 1 mL cell guardian (B-Bridge; Cupertino, CA, USA) per vial in liquid N_2_ and were used for subsequent experiments.

### Cell proliferation

Cells from lambs (n = 6 per treatment) at 1 day were separately plated in a 96-well plate (1,000 cells/100 μL maintenance media/well; n = 8 wells/lambs) and allowed to adhere for 24 hours, followed by serum deprivation for 24 hours, and then cultured in media with or without 10% FBS for 36 hours. To evaluate cellular proliferation, alamarBlue and 5-bromo-2'-deoxyuridine (BrdU) assays were performed. For alamarBlue assays, cells were rinsed twice (PBS), and a 1:10 dilution of alamarBlue indicator (Life Technologies): α-MEM at 100 μL/well was added. Plates were incubated at 37°C, 5% CO2 and fluorescence was detected at every hour for 24 hours using a Synergy2 plate reader (Biotek; Winooski, VT, USA) at 540/35 × 600/40 nm with a sensitivity of 54. For the BrdU assays, the Cell Proliferation ELISA BrdU (chemiluminescent) kit (Roche Diagnostics; Indianapolis, IN, USA) was used according to the manufacturer’s protocol.

### Gene expression

Gene expression was determined as previously described [6,19]. Briefly, MSC were cultured and seeded at a density of 100,000 cells per well and then allowed to attach and proliferate until they were 70 to 80% confluent. RNA from MSC was extracted using a Tri-Reagent (Qiagen, Valencia, CA, USA) and Qiagen Mini Kit according to the manufacturer’s protocol (Qiagen). Turbo DNA-Free kit (Ambion; Foster City, CA, USA) was used to remove residual genomic DNA. The concentration of RNA was determined using Nano-Drop (Thermo Scientific, Wilmington, DE, USA) and the quality of RNA was determined using Bioanalyzer (Agilent Technologies, Santa Clara, CA, USA). cDNA was synthesized from 300 ng total RNA (10 μL) using OligodT primer (1 μL; Ambion) and master mix containing 5.5 μL of 5x Buffer (Invitrogen), 1.0 μL dNTP (Promega; Madison, WI, USA), 2.0 μL DTT and 0.5 μL Superscript II making total reaction volume of 20 μL. Primers for genes involved in cell proliferation and lineage commitment were designed using Primer3 and NCBI BLAST for *Protein delta homolog 1*, *Msh homeobox 1*, *P2Y purinoceptor 2*, *P2Y purinoceptor 1*, and *P2Y purinoceptor 14*, validated as previously described [18,19], and synthesized (Integrated DNA Technologies, Coralville, IA, USA; S1 Table). Real-time RT-PCR was performed using iTAQ Universal SYBR Green Master Mix (Bio-Rad, Hercules, CA, USA) and the ABI 7900HT Fast Real-time PCR machine (Applied Biosystems, Foster City, CA). Each reaction included 5μL cDNA, 3 μL of nuclease-free water, 1 μL each of 10 nM forward and reverse primer and 10 μL of Sybergreen (BioRad) making a total volume of 20 μL. Real-time RT-PCR was performed using standard cycling conditions (Stage 1: 50°C for 2 min and 95°C for 10 min, Stage 2: 95°C for 15 s and 60°C for 1 min for 40 cycles, Stage 3: 95°C for 15 s and 60°C for 15 s with a 2% ramp to 95°C for 5 min). ^Δ^Ct values were obtained and used to calculate the ^ΔΔ^Ct values to determine relative gene expression [20] and Glyceraldehyde 3-phosphate dehydrogenase (GAPDH) mRNA expression was used as the internal control, as described previously [18].

### Mesenchymal stem cell mitochondrial respiration and glycolysis

Glycolytic and mitochondrial stress were evaluated using Seahorse XFe24 Extracellular Flux Analyzer (Seahorse Bioscience; North Billerica, MA) according to manufacturer’s protocol and previous publications [21,22] with the following modifications. Briefly, MSC were plated onto XFe24 cell culture plates at 30,000 cells per well and cultured for 48 hours in standard maintenance media according to protocols published in previous publications [18,21,22]. The XFe24 sensor cartridges were hydrated with Seahorse Bioscience XFe24 Calibrant (pH 7.4) and stored at 37°C for 24 hours [22,23].

For the Glycolysis Stress Test Assay, 2mM glutamine (Sigma-Aldrich; St Louis, MO, USA) was added to Seahorse XF Assay Media (Seahorse Bioscience). The media was warmed to 37°C and pH adjusted to 7.4 with 0.1 N NaOH and filter sterilized. The injection ports were loaded using the constant volume method described in the manufacturer’s protocol. The wells were rinsed with assay medium two times, and 525 μL of assay medium was added to each well, followed by a 60 minute incubation at 37°C without CO_2_. The plate was then loaded into the Seahorse XFe24 Extracellular Flux Analyzer. Standard operating procedure for Glycolysis Stress Test Assay for the machine was followed according to manufacturer’s protocol to obtain data.

For the Cell Mito Stress Test Assay, 1 mM pyruvate (Sigma-Aldrich, St Louis, MO), 2 mM glutamine (Sigma-Aldrich), and 10 mM glucose (Sigma-Aldrich) were added to the assay medium, warmed to 37°C and pH adjusted to 7.4 with 0.1 N NaOH. Oligomycin, FCCP, and rotenone/antimycinA were re-suspended with 630 μL, 720 μL, and 540 μL of prepared assay medium, respectively. The injection ports were loaded using the constant volume method described in the manufacturer’s protocol. The α-MEM maintenance medium was removed with a pipette, and the wells were rinsed twice with assay medium. The wells were then filled with 525 μL of assay medium, and the cell culture plates were incubated for 60 minutes at 37°C without CO_2_. The plate was then loaded into the Seahorse XFe24 Extracellular Flux Analyzer and standard operating procedure for Cell Mito Stress Test Assay for the machine was followed according to manufacturer’s protocol.

To account for the variation in final cell density, DNA content in individual wells of XFe24 cell culture plates were quantified using Macharey-Nagel NucleoSpin Tissue kits (Macharey-Nagel Inc, Bethlehem, PA, USA), according to manufacturer’s protocol. The extracellular acidification rate (ECAR) and oxygen consumption rate (OCR) measurements were adjusted for total DNA content in each well.

### Cell differentiation and staining

Cells from lambs (n = 6) at 1 day were separately plated at a density of 50,000 cells/well in 6-well (Greiner Bio-One) plates for osteoblast differentiation and 100,000 cells/well for adipocyte differentiation. Cells were induced to differentiate into osteoblasts with addition of media containing 10 nM Dexamethasone, 10 mM β-glycerophosphate, and 50 μg/mL ascorbic acid [18] or into adipocytes with addition of media containing 10% rabbit serum, 10 μg/mL Insulin, 0.5 mM 3-isobutyl-1-methylxanthine, and 200 μM Indomethacin) [24]. Cells were stained at day 0 and day 17 of culture in differentiation media with Alizarin red (Sigma-Aldrich) to quantify osteoblast differentiation or Oil Red O (Sigma-Aldrich) to quantify differentiation to adipocytes according to manufacturer’s protocol and as previously described [18]. For each animal, a total of three images were taken at day 0 and day 17 from three wells of 6-well plates using Axio Cam camera (Zeiss; Jena, Thuringia, Germany) mounted on an Axio Observer microscope (Zeiss) and the area stained was quantified using Image J (NIH) software [25].

### Statistical Analysis

For the analysis of proliferation, differentiation, metabolism, and gene expression data PROC MIXED procedure (SAS Inst. Inc, Cary, NC, USA) was used with dietary treatment as the fixed effect. Treatment mean comparisons were performed using LSMEANS statement and PDIFF option. For the proliferation assays, individual wells (n = 8) were averaged to obtain the value for each animal (n = 6/treatment). For the differentiation assays, the difference in the average of staining between day 0 and day 17 was calculated from three images from three separate wells and averaged together to obtain a value for an individual animal (n = 6/treatment). For the gene expression studies, mRNA from each individual animal was run in triplicate and the average of the ^Δ^Ct values were used for statistical analysis. For the Cell Mito Stress Test Assay and Glycolysis Stress Test Assay (n = 6/treatment) data were obtained by averaging the normalized value obtained from the individual wells to obtain a value per animal. For analyzing bone variables at 1 day or 3 months of age, maternal diet was treated as the fixed effect and a one-way ANACOVA was used, with body weight as a covariate (n = 6/treatment). Outlier analysis was performed and detected outliers were removed. Where appropriate, mean comparisons were made using least square means. Significant differences were determined to be significant at *P* ≤ 0.05 or a tendency at 0.05 < *P* ≤ 0.10.

## Results

### Bone variables

An effect of poor maternal diet was not observed for femur bone mineral content (*P* ≥ 0.26), bone area (*P* ≥ 0.40), bone density (*P* ≥ 0.30) and bone length (*P* ≥ 0.22) at day 1 and at 3 months of age (Table 1). Similar findings were observed for the tibia (data not shown).

**Table 1:** Bone variables^a^ in CON, RES and OVER lambs at 1 day and 3 months of age.

		Treatment^b^^,^^c^									
Item		CON		RES		OVER		SEM		*P*-Value	
Mineral content, g											
1 day of age		8.08		7.65		9.75		0.48		0.30	
3 months of age		36.69		35.08		39.37		2.34		0.26	
					
Area, cm^2^											
1 day of age		20.15		19.34		21.87		0.61		0.40	
3 months of age		49.69		47.44		50.10		2.07		0.67	
					
Density, g/cm^2^											
1 day of age		0.40		0.39		0.44		0.02		0.30	
3 months of age		0.74		0.74		0.78		0.03		0.47	
					
Length, cm											
1 day of age		11.53		11.36		11.90		0.15		0.90	
3 months of age		18.10		17.00		18.60		0.41		0.22	

^a^ Bone mineral content, bone area, and bone density are determined by dual-energy X-ray absorptiometry and bone length was determined using a caliper at 1 day and at 3 months of age for femur and tibia from right hind limb.

^b^ Means ± standard error of means (SEM) are reported

^c^ Ewe diet was based on the National Research Council (NRC) for energy requirements (total digestible nutrient) for pregnant ewes bearing twins, and began at day 30 ± 0.2 of gestation. Offspring from ewes fed a control (100% NRC, 1 day, n = 6; 3 months, n = 5), restricted (60% NRC, 1 day, n = 6; 3 months, n = 5), or over-fed (140% NRC, 1 day, n = 6; 3 months, n = 4) diet are referred to as CON, RES, and OVER, respectively.

### Mitochondrial respiration

**Fig 1:**
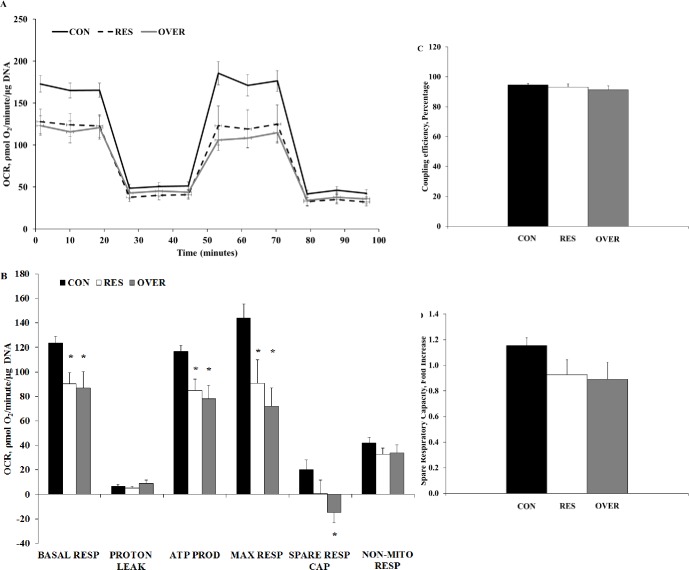
Poor maternal nutrition decreases the oxidative phosphorylation capacity of offspring mesenchymal stem cells. Mesenchymal stem cells were obtained from the offspring of control-fed (CON, n = 6), restricted-fed (RES, n = 6), and over-fed (OVER, n = 5) ewes at 1 day of age. Data are presented as mean ± standard error. * denotes *P* < 0.05. (A) Oxygen Consumption Rate (OCR), an indicator of oxygen-dependent mitochondrial ATP production, was measured when MSC were subsequently exposed to Oligomycin, FCCP, and Rotenone/Antimycin. (B) Calculation of basal respiration (BASAL RESP), proton leak (PROTON LEAK), ATP production (ATP PROD), maximal respiration (MAX RESP), spare respiratory capacity (SPARE RESP CAP), and non-mitochondria-derived respiration (NON-MITO RESP) of MSC, presented as mean ± standard error (C) Coupling efficiency (COUPLING EFF), represented as a percentage ± standard error (D) SPARE RESP CAP presented as a fold increase ± standard error.

In all treatment groups, OCR decreased with the injection of oligomycin, increased with the injection of FCCP, and decreased again with the injection of rotenone and antimycin A (Fig 1A). Compared with CON, basal respiration decreased by 29% and 31% in RES and OVER, respectively (127.4 ± 7.5, 90.2 ± 9.8, and 87.5 ± 8.5 ρmol O2/minute/μg DNA; CON, RES, and OVER, respectively; *P* ≤ 0.007; Fig 1B). Additionally, ATP production was reduced 30% and 36% in RES and OVER, respectively compared with CON (121.1 ± 6.1, 84.9 ± 11.1, and 77.7 ± 6.4 ρmol O_2_/minute/μg; CON, RES, and OVER, respectively; *P* ≤ 0.001; Fig 1B). Compared with CON, maximal respiration was reduced 39% and 55% in RES and OVER, respectively (149.29 ± 17.05, 90.64 ± 23.81, 67.93 ± 10.15 ρmol O_2_/minute/μg DNA; CON, RES, and OVER, respectively; *P* ≤ 0.03; Fig 1B). Spare respiratory capacity was reduced in OVER compared with CON, while RES was not different from CON and OVER (21.9 ± 10.8, 0.47 ± 12.3, and -19.56 ± 9.24 ρmol O_2_/minute/μg DNA; CON, RES, and OVER, respectively; *P* ≤ 0.06; Fig 1B). An effect of poor maternal nutrition was not observed for proton leak, non-mitochondrial respiration, coupling efficiency or fold increase of spare respiratory capacity (*P* ≥ 0.20; Fig 1B, 1C and 1D).

### Glycolytic stress

**Fig 2:**
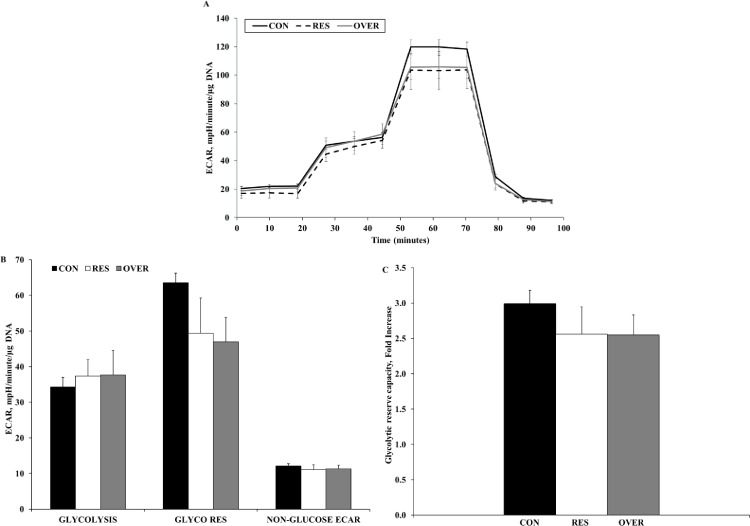
Glycolysis Stress Assay for MSC. Mesenchymal stem cells were obtained from the offspring of control-fed (CON, n = 6), restricted-fed (RES, n = 6), and over-fed (OVER, n = 5) ewes at 1 day of age. Data are presented as mean ± standard error. (A) Extracellular Acidification Rate (ECAR), an indication of glycolysis, was measured when MSC were subsequently exposed to glucose, oligomycin, and 2-deoxyglucose. (B) Calculation of basal glycolysis (GLYCOLYSIS), glycolytic reserve (GLYCO RES), and non-glucose-derived ECAR (NON-GLUCOSE ECAR) of MSC presented as mean ± standard error. (C) Glycolytic Reserve Capacity (GLYCO RES CAP) represented as fold increase of MSC. Data are presented as mean ± standard error.

The extracellular acidification rate (ECAR) increased in all treatment groups after MSC were exposed to glucose, and increased further after the injection of oligomycin and decreased with the injection of 2-DG decreased ECAR in all treatment groups (Fig 2A). An effect of poor maternal nutrition was not observed for any of the variables evaluated for glycolytic stress of MSC, including glycolysis, glycolytic capacity, non-glucolytic acidification rate, and glycolytic reserve capacity (*P* ≥ 0.18; Fig 2B and 2C).

### Cell proliferation

**Fig 3:**
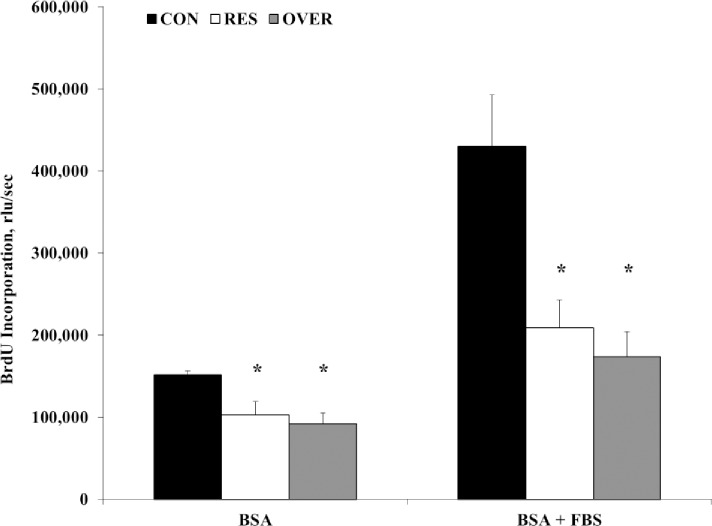
Poor maternal nutrition reduces proliferation of offspring mesenchymal stem cells. MSC were obtained from the offspring of control-fed (CON, n = 5), restricted-fed (RES, n = 6), and over-fed (OVER, n = 6) ewes at 1 day of age. Cells were cultured in the absence [bovine serum albumin (BSA)] or presence [BSA + fetal bovine serum (FBS)] of serum and proliferation was determined by 5-bromo-2'-deoxyuridine (BrdU) assay. Data are presented as mean ± standard error. *P < 0.01 rlu/sec = relative fluorescence units/sec.

In presence of serum, MSC proliferation was reduced 60% and 51%, in OVER and RES, respectively compared with CON (429,780 ± 79,964; 208,902 ± 39,020; 173,498± 35,631 relative light units (rlu)/sec; CON, RES, and OVER, respectively; *P* ≤ 0.009; Fig 3). Similarly, in the absence of serum, there was a reduction in MSC proliferation of 39% and 32% in the OVER and RES, respectively compared with CON (151,767 ± 5,657; 102,756 ± 18,725; 91,847 ± 15,142 rlu/sec; CON, RES, and OVER, respectively; *P* ≤ 0.04; Fig 3). A similar magnitude and direction of reduction in proliferation was also observed using alamar blue assay (data not shown).

### Cell differentiation

**Fig 4:**
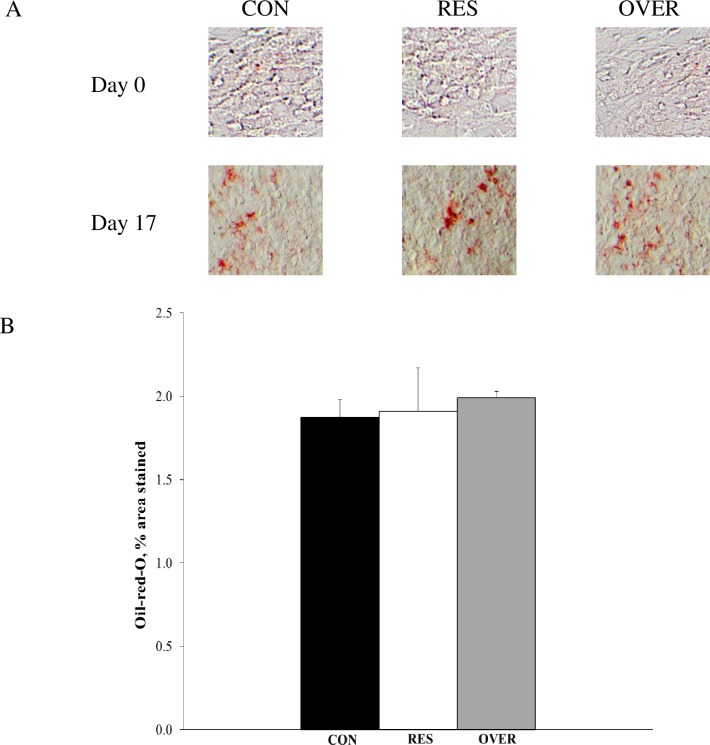
Impact of poor maternal nutrition on differentiation of mesenchymal stem cells into adipocytes. Mesenchymal stem cells were obtained from the offspring of control-fed (CON, n = 6), restricted-fed (RES, n = 6), and over-fed (OVER, n = 6) ewes at 1 day of age. Cells were plated in 6-well plates and differentiation was induced by addition of adipocyte differentiation media. ImageJ software was used to quantify staining (A) and data are presented as difference of mean values observed at day 17 and day 0 ± standard error (B).

**Fig 5:**
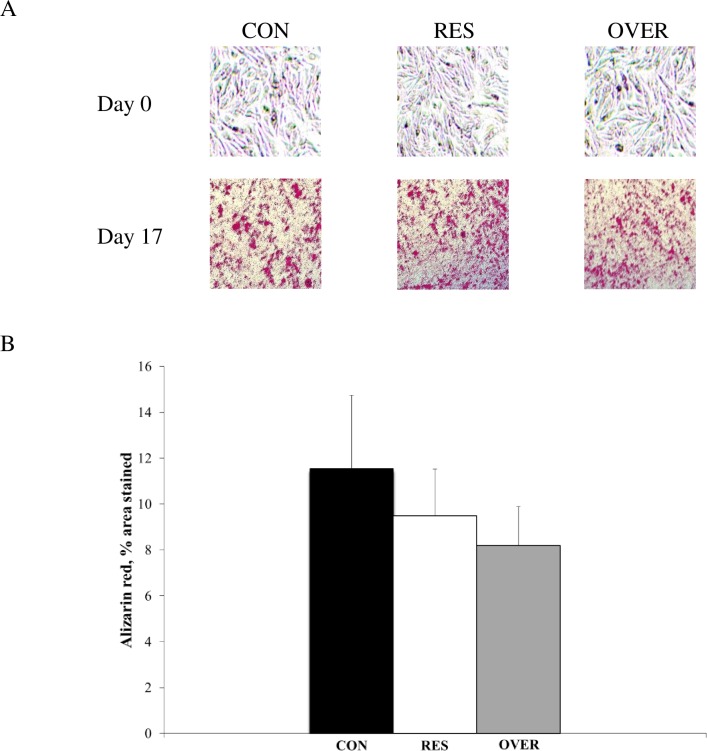
Impact of poor maternal nutrition on differentiation of mesenchymal stem cells into osteoblast Mesenchymal stem cells were obtained from the offspring of control-fed (CON, n = 6), restricted-fed (RES, n = 6), and over-fed (OVER, n = 6) ewes at 1 day of age. Cells were plated in 6-well plates and differentiation was induced by addition of osteoblast differentiation media. ImageJ software was used to quantify staining (A) and data are presented as difference of mean values observed at day 17 and day 0 ± standard error (B).

Mesenchymal stem cells were successfully differentiated into adipocyte and osteoblast cells as demonstrated by an 86% and 94% increase in area stained, respectively. An effect of poor maternal nutrition on the differentiation of MSC into adipocytes (*P* > 0.33) was not observed in 1 day offspring (Fig 4A and 4B). Also, an impact of poor maternal nutrition on the differentiation of MSC into osteoblasts was not observed (*P* > 0.55) in 1 day offspring (Fig 5A and 5B).

### Gene expression data

In ovine MSC from 1 day, the expression of P2Y1 tended to be reduced (*P* = 0.08; Table 2) 2.7 fold by the maternal diet in OVER compared with CON. We did not observe an effect (*P* ≥ 0.14; Table 2) of poor maternal nutrition on the expression of genes *Pref-1*, *P2Y14*, *P2Y2* or *Msx-2*.

**Table 2:** Gene expression^a^ of key transcription factors involved in proliferation and differentiation of mesenchymal stem cells in CON, RES and OVER offspring at 1 day of age.

					
		Treatment^b^^,^^c^			
Gene	CON	RES	OVER	SEM	*P*-value
*Pref-1*	1.02	1.12	1.35	0.15	0.25
*Msx2*	1.21	1.14	0.80	0.40	0.21
*P2Y2*	1.08	0.98	0.91	0.16	0.75
***P2Y1***	**1.22**	**0.85**	**0.45**	**0.16**	**0.08**
*P2Y14*	1.13	0.70	0.68	0.10	0.14

^a^Relative to CON, means ± standard error of means (SEM) are reported

^b^ Gene expression was determined by real time RT-PCR for MSC collected from offspring at 1 day of age.

^c^ Ewe diet was based on the National Research Council (NRC) for energy requirements (total digestible nutrients) for pregnant ewes bearing twins, and began at day 30 ± 0.2 of gestation. Offspring from ewes fed a control (100% NRC, 1 day, n = 6), restricted (60% NRC, 1 day, n = 6) or over-fed (140% NRC, 1 day, n = 6) diet are referred to as CON, RES, and OVER, respectively.

## Discussion

Self-renewal capacity is a key feature of stem cells, which helps to maintain a pool of these cells in the organism [15,26]. Additionally, maintaining these cells is essential for the development and maintenance of various tissues [15,27]. Specifically, MSC are critical for tissue growth, maintenance, and repair from fetal development through adulthood. Mesenchymal stem cells are key components of the bone marrow niche, which is responsive to nutrient, hormonal, and metabolic changes in the whole body [16]. Since these cells were cultured in controlled in vitro conditions, the 50% reduction in proliferation of MSC from both RES and OVER offspring suggests maternal programming of the offspring MSC. Furthermore, our findings of reduced MSC proliferation in lambs are similar to findings in rodents [11]. Mesenchymal stem cells of offspring from mothers fed a low protein diet exhibited reduced proliferation and ability to differentiate into bone forming cells (osteoblasts) when collected from pups 8 weeks of age [11]. This reduced proliferation of MSC may affect the development and maintenance of tissues such as bone, fat, and muscle by reducing the stem cell pool [5,14]. We previously reported that in this study that maternal nutrient restriction reduced the backfat thickness in the offspring [13], therefore it is possible that alterations to cellular proliferation of MSC may have altered the establishment of this adipose depot. In addition to changes in proliferation, changes to cellular metabolism and function may be mediating some the effects of poor maternal nutrition on offspring growth and development.

Previous studies have found that metabolism is a key determinant of whether a cell proliferates, differentiates, or remains quiescent [28–30]. A recent study conducted by Lyublinskaya and colleagues found that reactive oxygen species (ROS) are required for MSC to initiate proliferation [31]. Additionally, reduced level of basal oxidative phosphorylation will lead to a reduced ROS in MSC [21,32]. Therefore, the reduced level of oxidative phosphorylation in RES and OVER in the current study may be contributing to reduced proliferation in RES and OVER, through a reduction in ROS.

Bone marrow-derived MSC are suited for a low-oxygen niche that promotes the maintenance of the undifferentiated state [33]. However, the MSC used in the culture and assay conditions are no longer in this hypoxic environment and therefore the increased oxygen availability should increase oxygen utilization [33]. mTOR is one such signaling pathway that upregulates metabolism in response to the sensing of increased oxygen and nutrient availability [33]. Therefore, it is expected that cells maintain a greater metabolic state under increased oxygen availability. Under normoxia, the MSC from RES, OVER, and CON all experienced comparable rates of glycolysis, which is likely because oxygen is not involved in glycolysis.

There is an important balance between proliferation (and therefore increased metabolic activity) and stem cell longevity. When proliferation is restricted, stem cell longevity is extended through decreased cellular aging, quiescence, and stem cell exhaustion [33]. Alternately, increased metabolic activity promotes proliferation but can cause cellular injury, especially in stem cells, through ROS and oxidative stress. We found that MSC from RES and OVER had both reduced proliferation and reduced oxidative phosphorylation, compared with CON. Oreffo et al. [2013] also demonstrated that proliferation of MSC was reduced in offspring of protein-restricted ewes. Therefore, it is possible that MSC from RES and OVER are programmed to limit oxidative phosphorylation which limits ROS and preserves stem cell longevity, as the animal is preparing for birth into an anticipated unfavorable environment. When considering cellular metabolism, the function of the mitochondria in the cell also needs to be taken into consideration.

Mitochondria have an important role in the maintenance and differentiation of stem cells [21]. During differentiation of MSC into specific cell types, such as osteoblasts or adipocytes, there is a critical shift from glycolytic to aerobic metabolism [34]. During this shift, there is a need for upregulation of mitochondrial function. In the present study, to gain insight into the effect of maternal nutrition on MSC energy metabolism, we determined mitochondrial respiration and glycolysis capacities of MSC. The reduced basal respiration and ATP production in MSC of both RES and OVER offspring suggest that the MSC have reduced ability to upregulate ATP production during energetic deficits. Additionally, as these findings were observed when cultured in controlled in vitro conditions, they suggest potential programming of these stem cells resulting in impaired differentiation potential. Consistent with reduced ATP production, we observed reduced expression of PGC-1alpha in the muscle of OVER offspring at birth [35]. This is consistent with the hypothesis that reduced mitochondrial activity in MSC impairs development of tissue, such as muscle. Overall, the changes in the metabolic profile of MSC observed in our study can potentially lead to the development of whole body metabolic dysfunction, and associated metabolic diseases such as insulin resistance, obesity, oxidative stress, and mitochondrial dysfunction in sheep [3].

Adequate bone development is important due to its critical role in structural support and framework for the body in addition to its role in protecting soft tissues, maintaining mineral balance, and hematopoiesis [36]. There is evidence that the environment can alter bone development [14]; however previous reports vary depending on species, model, and age. To date most reports of altered bone mass due to poor maternal nutrition during gestation were observed in adult offspring [9,37,38]. In a rat model, maternal protein deprivation during pregnancy modified growth-plate morphology and negatively impacted bone composition, length, and mechanical strength in offspring [11,39]. Epidemiological studies have also associated low birth weight with reduced bone mineral content and increased risk of osteoporosis later in life for humans [40,41]. The absence of an effect of maternal diet on offspring bone at birth and 3 months of age in the current study may be due to the early time points evaluated. During fetal development, in addition to critical organs such as the brain and heart, bone receives priority for nutrients partitioning and unless a severe restriction occurs, it is unlikely that changes in bone development and mineralization will be detected at this time point. However, due to the loss of bone that naturally occurs in adults and aging humans and animals, it is possible that maternal diet programs the offspring for a similar accelerated bone loss that is observed in adult offspring [14]. Consistent with the nutrient priority during development, we and others have observed impaired muscle and fat development during the early postnatal period of poorly fed mothers [6,9,13,35,37]. Additionally, restricted feeding during gestation impaired satellite cell function and altered mRNA expression in muscle of offspring [12]. These findings are consistent with the nutrient priority to bone and organs during early development compared with muscle and adipose. However, it is possible, that poor maternal nutrition may be altering the function of the MSC during gestation and therefore impacting the role of these cells in establishing key tissues, such as the muscle and adipose. To better understand this potential involvement, we are currently evaluating the effects of poor maternal nutrition on MSC during fetal development.

Based on the knowledge that many tissues of mesenchyme origin are also developed and maintained during postnatal growth through the pool of MSC, we further investigated the effects of maternal diet on the function of offspring MSC. Specifically, based on the recent reports that changes in gene expression and maternal diet can alter the lineage commitment of MSC [14,42], we determined if maternal diet altered the ability of MSC to differentiate into adipocytes and osteoblasts. Consistent with bone phenotype, we did not observe an effect of diet on differentiation into osteoblasts or adipocytes. The absence of an effect of maternal diet on the differentiation of MSC might be due to the early time point at which the samples were collected or variation from the methods used for the estimation of differentiation. Further studies are warranted at different time points to evaluate the effect of poor maternal nutrition on differentiation ability of MSC.

Several key transcription factors and regulatory genes determine the ability of MSC to proliferate and differentiate of MSC [43]. Of particular interest are the purinergic receptors, which have critical roles in committing MSC to differentiate into adipocyte or osteoblasts lineages [44,45]. *Purinergic receptor 2Y* is involved in the proliferation of MSC, as well as the differentiation and migration of MSC [45]. Therefore, the reduced expression of *Purinergic receptor 2Y 1* in the OVER group could be a potential mechanism contributing to the reduced proliferation. However, further studies to explore its role in MSC response to maternal diet, including changes in protein expression, are needed. Additional analysis is needed to evaluate global changes in gene expression to further elucidate the mechanisms by which maternal diet alters offspring MSC function.

In conclusion, the findings in the current study demonstrated the programming effects of poor maternal nutrition during gestation on offspring stem cell function and metabolism. Furthermore, this study demonstrates that alterations to the cellular bioenergetics of MSC as a possible mechanism for the reduced MSC proliferation and more importantly previously reported impaired muscle, bone, and adipose development. The molecular mechanisms by which these changes occur in MSC are currently unknown and warrant further investigation.

## Supporting Information

S1 Table Primer Sequences. Primers were designed using Primer3 and NCBI BLAST synthesized by Integrated DNA Technologies.
(DOCX)
