# Supplementary material for: Effects of Poor Maternal Nutrition during Gestation on Bone Development and Mesenchymal Stem Cell Activity in Offspring
Source: PLoS One. 2016 Dec 12;11(12):e0168382. doi: 10.1371/journal.pone.0168382 (PMC5152907; doi:10.1371/journal.pone.0168382)
Supplement: S1 Table — Primers were designed using Primer3 and NCBI BLAST synthesized by Integrated DNA Technologies. (DOCX) [file pone.0168382.s001.docx]

**S1 Table. Primer sequences**

| **Primers** | **Forward sequence (5’ – 3’)** | **Reverse sequence (3’ – 5’**) |
| --- | --- | --- |
| *Protein delta homolog 1* | CGG GTT CTC AGG AAA GGA TT | CAG CTG TTG GTC ACG ATC TC |
| *Msh homeobox 1* | ACA CAA GAC CAA TCG GAA GC | GGG GAG CAC AGG TCT ATG TG |
| *P2Y purinoceptor 2* | CCT CCC TGC CGC TGC TGG TT | TCT GTG GCG GGC TTG GCA TC |
| *P2Y purinoceptor 1* | TCC GGA AAA ACA AAA CCA TC | CTC CTC AGA GGC GAA TTG TC |
| *P2Y purinoceptor 14* | TGA TCC TGA CCA ATC GGA AT | TGC CCA CAA AGA TGT AGC TG |

Primers were designed using Primer3 and NCBI BLAST synthesized by Integrated DNA Technologies.
